# A Model for Predicting Clinical Prognosis in Patients with WHO Grade 2 Glioma

**DOI:** 10.1155/2022/2795939

**Published:** 2022-11-26

**Authors:** Qi Gao, Huandi Zhou, Guohui Wang, Zhenghui Ma, Jiayuan Li, Hong Wang, Guozhu Sun, Xiaoying Xue

**Affiliations:** ^1^Department of Radiotherapy, The Second Hospital of Hebei Medical University, Shijiazhuang 050000, Hebei Province, China; ^2^Department of Central Laboratory, The Second Hospital of Hebei Medical University, Shijiazhuang 050000, Hebei Province, China; ^3^MD Anderson Cancer Center, University of Texas Hospital, Houston, Texas 77030, USA; ^4^Department of Neurosurgery, The Second Hospital of Hebei Medical University, Shijiazhuang 050000, Hebei Province, China

## Abstract

**Objectives:**

Although patients with grade 2 glioma have a relatively better prognosis and longer survival than those with high-grade glioma, there are still a number of patients with disappointing outcomes. In order to accurately predict the prognosis of patients, relevant risk factors were included in the analysis to establish a clinical prediction model so as to provide a basis for clinically individualized treatment.

**Methods:**

A retrospective study was conducted in patients diagnosed with grade 2 glioma. Data including clinical features, pathological type, molecular classification, neuroimaging examination, treatment, and survival were collected. The data sets were randomly assigned, with 80% of the data used for model building and 20% for validation. Cox proportional hazard regression analysis was used to construct the model using important risk factors and present it in the form of a nomogram. The nomogram was evaluated a using *C*-index and calibration chart.

**Results:**

A total of 160 patients were enrolled in this analysis, including 128 in the training group and 32 in the validation group. In the training group, eight important risk factors including preoperative KPS, the first presenting symptom, the extent of resection, the gross tumor size, 1p19q, IDH, radiotherapy, and chemotherapy were identified to construct the model. The *C*-index of the training group and the validation group was 0.832 and 0.801, respectively, indicating that the model had good prediction ability. The calibration charts of the two groups were drawn respectively, which showed that the calibration line and the standard line had a good consistency, which suggested that the model-predicted risk had a good consistency with the actual risk.

**Conclusions:**

Based on the data of our center, a nomogram prediction model with eight variables has been established as an off-the-rack tool and verified its accuracy, which can guide clinical work and provide consultation for patients.

## 1. Introduction

Adult-type diffuse gliomas are the most common neurogenic tumor in adult primary brain tumors. The 2016 edition of the World Health Organization (WHO) classification of central nervous system (CNS) tumors included molecular typing as the classification criteria for the first time, which ended the classification mode that had relied solely on histopathology for nearly a century [1]. The WHO grade 2 glioma accounts for 5% of all primary brain tumors [2]. Although patients with grade 2 glioma have a relatively better prognosis and longer survival than those with high-grade glioma, there are still a number of patients with disappointing outcomes. Such tumors bring great challenges to clinical management, which requires us to determine the best treatment strategy according to the specific situation of each patient. The premise for achieving this goal is to accurately predict the prognosis of patients, so it is particularly important to establish a prognostic prediction models for grade 2 glioma in this study.

As a reliable clinical tool, the nomogram has been widely used in clinical decision making, and it can help physicians predict survival, decide on individualized treatment plans and determine follow-up times [3]. The most obvious advantages of nomogram are its accurate predictability, accessibility, and intuitiveness [4].

Therefore, the purpose of the study is to construct a validated nomogram that could predict the survival of patients with WHO grade 2 glioma using relevant clinical variables. All patient data evaluated in this study were from that institution.

## 2. Materials and Methods

### 2.1. Study Population

The study population was selected from adult patients with WHO grade 2 glioma confirmed by pathological findings in our center from January 2010 to December 2019. We identify and document risk factors that influence patient prognosis, including sex, age, preoperative Karnofsky Performance Status (KPS) score, first presenting symptom, extent of resection, location of the tumor, gross tumor size, pathological type, 1p19q, IDH, radiotherapy, and chemotherapy. Patients enrolled in the criteria were randomly divided into the training group and the validation group in a ratio of 4 : 1. The training group was used to build the model, and the validation group was used to verify the model.

### 2.2. Operational Definition

The KPS score is the most commonly used functional status rating scale for adults, and it is divided into 10 stages on a 100-point scale [5]. It was divided into two groups, including KPS scores ≥80 and <80 groups. Neurosurgeons assess tumor size and location based on brain MRI. Deep tumors were defined as those that were difficult to reach surgically. Postoperative residual tumor size was determined by postoperative MRI or contrast-enhanced brain CT. Gross total resection was defined as complete resection of the tumor or the neurosurgeon's opinion that only <5% residual tumor was found on postoperative imaging. Nontotal resection was defined as a residual tumor of more than 5%. A biopsy was defined as a procedure that was performed only for pathological diagnosis and did not attempt to remove the tumor. Temozolomide was the chemotherapy regimen used in all our enrolled cases. Concurrent chemoradiotherapy is the main treatment option for most patients, including patients with partial resection or biopsy. A small number of patients choose chemotherapy alone or radiotherapy alone because they cannot tolerate high-intensity treatment, such as low KPS score and contraindication of chemoradiotherapy.

The follow up was completed until December 2020 including death or survival and cause of death.

### 2.3. Statistical Analyses

The variables in the training group and the validation group were described, and the chi-square test was used for statistical analysis of the variables to compare whether the differences were statistically significant. Kaplan–Meier survival analysis was used to calculate the 3-year and 5-year survival rates of patients and draw survival curves, which can intuitively show the influence of various variables on the prognosis of patients. Then, the log-rank test was used to compare whether there was a statistical difference in the influence of each variable on prognosis. In the training group, all risk factors identified and collected were used as independent variables to conduct univariate cox regression analysis one by one. The variables with *P* < 0.05 in univariate analysis were used as independent variables to enter multivariate cox regression analysis and identified as independent risk factors. The hazard ratio (HR) and 95% confidence interval (CI) were calculated. *P* values <0.05 were considered to indicate statistical significance.

### 2.4. Construction and Evaluation of the Models

The model is presented in the form of the nomogram. Use the nomogram function in the RMS package of the R language to draw the nomogram for predicting 3-year and 5-year survival using the variables screened out by deployment as independent risk factors. Certain variables are not screened out, but these variables were kept in the multivariable models due to clinical importance. The validation group data will be used for external validation at nomogram to evaluate the performance of the model. The *C*-index and calibration chart are reliable methods to verify the accuracy of nomograms. The *C*-index function in R language is used to calculate the *C*-index value of the model. The higher the number and the closer it is to 1, the better the prediction ability of the model. Then, the calibration chart is drawn, and the better the coincidence between the calibration line and the standard line, the better the prediction ability of the model.

## 3. Results

### 3.1. Patient Baseline Characteristics

A total of 160 patients were enrolled in this analysis, including 128 in the training group and 32 in the validation group. The clinical data of all patients were stratified according to the designated set. There were no statistically significant differences in clinical baseline characteristics between the training group and the validation group (see Table 1).

### 3.2. Survival Analyses

**Figure 1:**
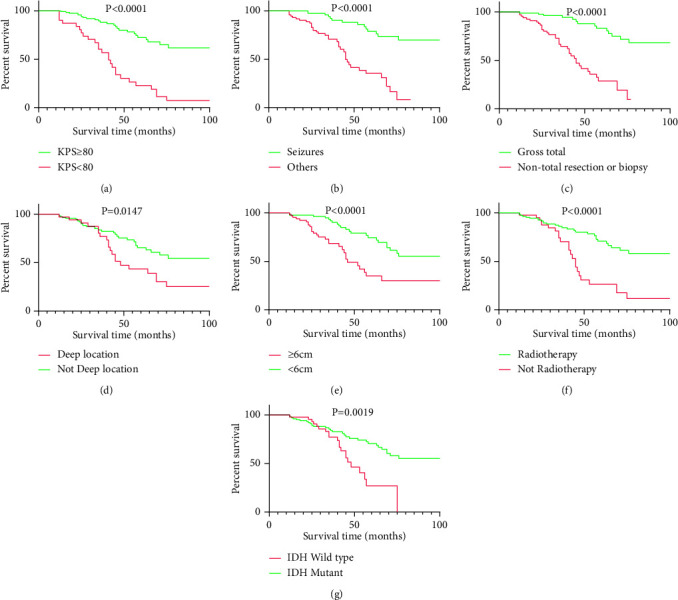
The survival curves of variable with *P* value less than 0.05 were tested by the log-rank. (a) Preoperative KPS. (b) First presenting symptom. (c) Extent of resection. (d) Location of the tumor. (e) Gross tumor size. (f) Radiotherapy. (g) IDH.

The median follow-up time of patients was 37 months, and Kaplan–Meier survival analysis showed that the 3-year and 5-year survival rates were 80.9% and 57.4%, respectively, with a median survival of 71 months. The log-rank test showed that preoperative KPS, first presenting symptom, extent of resection, location of the tumor, gross tumor size, radiotherapy, and IDH were correlated with survival prognosis and that the survival difference was statistically significant (see Figure 1).

### 3.3. Univariate and Multivariate Analyses

**Figure 2:**
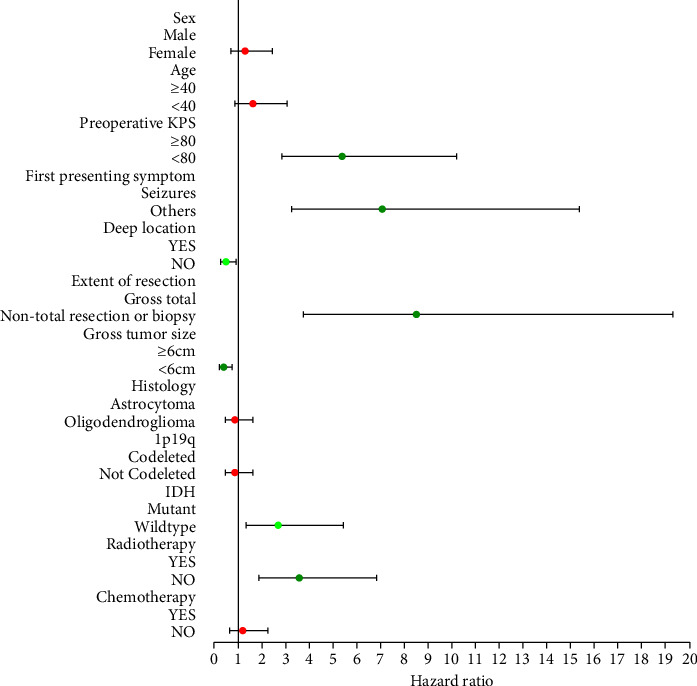
Univariate regression analysis of training group.

**Figure 3:**
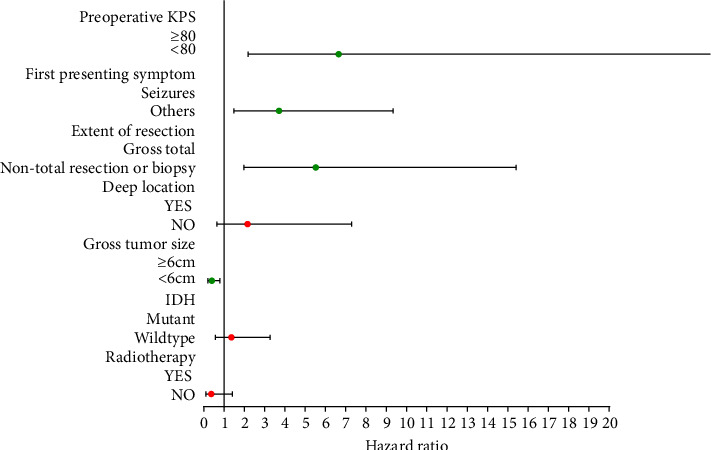
Multivariate regression analysis of training group.

In the training group, cox proportional hazard regression analysis was used for univariate and multivariate analyses (see Table 2). Univariate analyses revealed that preoperative KPS, the first presenting symptom, the extent of resection, the location of the tumor, the gross tumor size, the IDH, and radiotherapy significantly affected overall survival (see Figure 2). Then, these variables were treated as independent variables to enter multivariate cox regression analysis. Finally, multivariate analysis showed that preoperative KPS, the first presenting symptom, the extent of resection, and the gross tumor size were four independent prognostic factors (see Figure 3).

### 3.4. Nomogram Construction

**Figure 4:**
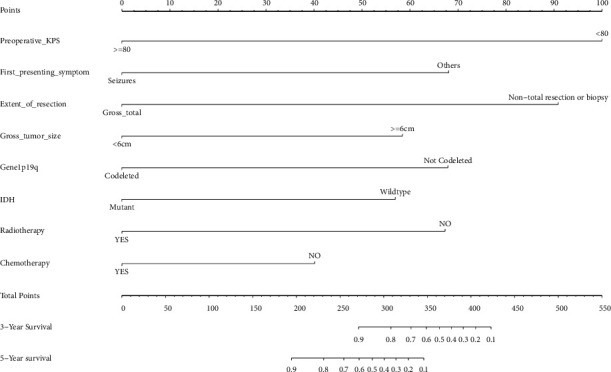
Nomogram of predicting 3-year and 5-year survival.

Four independent prognostic factors selected by multivariate analysis were used as the predictors. Molecular typing and treatment were not found to be statistically significant, but these variables were kept in the multivariable models due to their clinical importance. Ultimately, eight prognostic factors were used to construct the model and present it in the form of the nomogram (see Figure 4). In the nomogram, different scores are given according to the status of each factor, and then, all scores are added to get a total score, and the corresponding 3-year or 5-year survival rate is obtained based on the total score. The nomogram shows that preoperative KPS has the strongest correlation with prognosis. The survival rate of each patient can be easily and intuitively calculated by the cumulative scores of each variable (see Table 3).

### 3.5. Nomogram Validation

**Figure 5:**
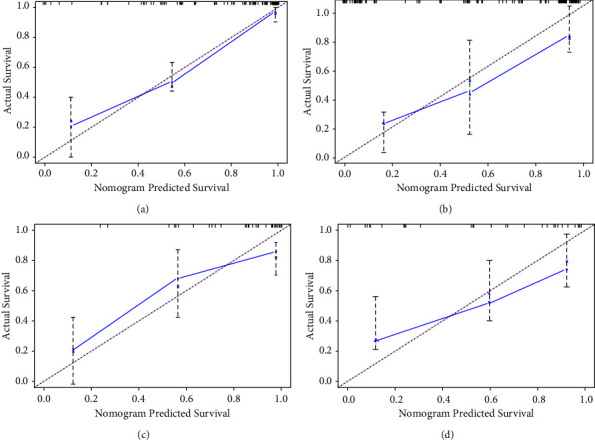
(a) Three- and (b) 5-year nomogram calibration curves for internal calibration plots of overall survival. (c) Three- and (d) 5-year nomogram calibration curves for external calibration plots of overall survival.

The internal validation using the nomogram to predict survival had a *C*-index of 0.832 (95% CI: 0.786–0.872), and the external validation of the validation group data applied to the nomogram had a *C*-index of 0.801 (95% CI: 0.735–0.912). The results show that the model has a good performance in prediction. Whether it is internal validation or external validation, as can be seen from the calibration chart, the actual prediction curve of the model has a high degree of coincidence with the validation curve, indicating a high degree of consistency between the predicted risk and the actual risk, and the actual observed values and predicted values of the nomogram show good consistency in both the training group and the verification group (see Figure 5).

## 4. Discussion

Due to the large heterogeneity and the large difference in prognosis and survival time of grade 2 glioma, it is of great significance to predict the survival and prognosis of this type of glioma for clinical diagnosis and treatment. With the development of various clinical studies and the support of high-level clinical evidence, especially the clinical application of tumor molecular typing, the development of personalized diagnosis and treatment has provided a strong impetus. As a graphical scoring tool, the nomogram is often used in various statistical prediction models. It can calculate the probability of survival according to the individual characteristics of patients, and it has become an important part of modern medicine [4]. The study collected clinical data from 160 patients with grade 2 glioma, screened predictors by cox proportional hazard regression analysis and deployment, and then developed a clinical nomogram. It can predict survival rates based on the clinical characteristics of patients and have been proven to have good predictive accuracy.

The present nomogram consists of 8 prognostic factors: preoperative KPS, the first presenting symptom, the extent of resection, the gross tumor size, 1p19q, IDH, chemotherapy, and radiotherapy. Good preoperative functional status, seizures as the main initial symptoms, small tumor size, complete resection of the tumor, 1p19q-codeleted, IDH mutant, postoperative effective radiotherapy, and chemotherapy were associated with improved survival for patients with grade 2 glioma.

The nomogram revealed that preoperative KPS was most strongly associated with the prognosis. After tumor diagnosis, the patient's functional status is the primary consideration for clinicians to decide which treatment method to take. The KPS score is a scale for evaluating functional impairment. The lower the score, the worse the quality of life, and the less likely it is to be treated aggressively, and thus, affect the patient's prognosis.

The grade 2 glioma is the main type of low-grade glioma (LGG). Symptoms of LGG vary depending on the location and size of the tumor, mainly due to the mass effect [6]. Seizure is the most common clinical symptom, occurring in more than 90% of LGG patients at some stage of the disease, mostly in the frontal lobe of patients with oligodendroglioma [7]. In the largest retrospective study published by Pallud et al. seizure was found to be an independent predictor of overall survival [8], which is consistent with our findings. Seizure at the onset in LGG patients predict the possibility of continuous occurrence of postoperative seizure and are related to prognosis. In patients with intact nervous system, manifestations associated with seizure are related to better prognosis, which may be related to early diagnosis of patients by timely medical treatment. The occurrence of headache, paresthesia, and other nonepileptic symptoms are often easily ignored by patients.

Total tumor resection can minimize tumor load, reduce the risk of transition to higher-grade gliomas, and improve the efficacy of subsequent adjuvant therapy [9]. However, it is difficult to make a definitive diagnosis if a small excision or biopsy is performed [10]. A large number of studies suggest that the excision range has a positive effect on the natural history of the disease, significantly delays the time to malignant progression, and is an independent predictor of survival regardless of age, preoperative tumor volume, and functional status [11, 12].

Surgery has always been a focus in the treatment of grade 2 gliomas [13]. If the tumor volume is large, it is easy to damage important functional areas during surgical resection and cause new neurological dysfunction, thus reducing the surgeon's willingness to perform radical resection of such tumors. Therefore, for patients with large tumors, the surgical resection scope needs to be weighed, and sometimes it is difficult to achieve radical resection, thus affecting the prognosis of patients [14]. The national comprehensive cancer network guidelines have identified tumor size as an important independent prognostic factor, and our study confirmed that tumor size ≥6 cm is a negative prognostic factor.

For high-risk patients with grade 2 glioma, tumor recurrence or progression may occur even after total surgical resection, thus, adjuvant therapy with radiotherapy and chemotherapy is required. Radiotherapy for patients with grade 2 gliomas has been controversial. The European Organization for Research and Treatment of Cancer 22845 trial observed the efficacy of postoperative radiotherapy with LGG [15]. The study set up the early postoperative treatment group and the advanced postoperative treatment group, and the results showed that the difference in median progression-free survival (PFS) between early radiotherapy and delayed radiotherapy was statistically significant, while the difference in median overall survival (OS) was not statistically significant. Therefore, it is recommended that radiotherapy should be performed after disease progression for low-risk patients, while early radiotherapy should be considered for high-risk patients. Although the results showed that radiotherapy alone did not prolong the OS, but had some efficacy in alleviating edema, intracranial pressure, and symptoms of focal neurological deficits.

The Radiation Therapy Oncology Group 9802 trial is one of the first to evaluate radiotherapy combined with chemotherapy for high-risk LGG [16]. The results showed that the addition of chemotherapy to radiotherapy had a better OS and PFS and a lower risk of recurrence compared with radiotherapy alone. Temozolomide has been widely used in clinical practice due to its oral administration, low toxicity, and easy access. Numerous studies have shown that the advantages of adjuvant therapy are mostly reflected in patients with high-risk factors [17–20], so it is necessary to screen patients rationally, and the high-risk predictors in our study can provide a reference. In our study, the difference in prognosis between patients receiving adjuvant therapy and those not receiving adjuvant therapies did not reach statistical significance, which may be caused by the inclusion of some low-risk postoperative patients due to the year limitation.

Molecular phenotypes even outweigh histopathology in the new classification of CNS tumors, largely due to the mutated status of IDH. At the same time, most studies have shown that LGG patients with the IDH mutation have a better prognosis [21, 22]. Based on this, the definitive guidelines have clearly indicated that IDH is an important indicator for the molecular typing of gliomas. Our study also confirmed that IDH mutation status is a risk factor for grade 2 glioma. Therefore, it is recommended that patients with grade 2 glioma should be tested for IDH status after surgery to guide subsequent treatment. Studies have shown that oligodendroglioma patients characterized by an IDH mutation and 1p19q-codeleted have a better prognosis than astrocytoma patients [23, 24]. Therefore, detection of 1p19q-codeleted is of great significance in the diagnosis of oligodendroglioma and the prognosis of patients. Compared with patients with 1p19q not codeleted, patients with the IDH mutation combined with 1p19q-codeleted have the best prognosis.

The survival of grade 2 glioma was affected by many factors. The model constructed in this study manages these risk factors in a unified manner, allowing clinicians to calculate the survival probability of patients conveniently and quickly, thus guiding further treatment. The validity of the model has been verified by identification and correction. Finally, this study still has some limitations. As a retrospective study, the influence of bias and confounding factors cannot be completely excluded. Multivariate analysis was used to adjust and solve this limitation as much as possible [25]

## 5. Conclusions

Based on the data of our center, a nomogram prediction model with eight variables has been established as an off-the-rack tool and verified its accuracy, which can guide clinical work and provide consultation for patients.

## Figures and Tables

**Table 1:** Baseline characteristics of the study population.

Variables	Training groups (*n* = 128)	Validation groups (*n* = 32)	*P* values
*Sex*			0.380
Male	75	16	
Female	53	16	

*Age (years)*			0.461
≥40	79	22	
<40	49	10	

*Preoperative KPS*			0.361
≥80	94	26	
<80	34	6	

*First presenting symptom*			1.000
Seizures	64	16	
Others	64	16	

*Extent of resection*			0.750
Gross total	72	19	
*Nontotal resection or biopsy*	56	13	

*Deep location*			0.570
Yes	30	6	
No	98	26	

*Gross tumor size*			0.068
≥6 cm	53	19	
<6 cm	75	13	

*Histology*			0.102
Astrocytoma	52	8	
Oligodendroglioma	76	24	

*1p19q*			0.102
Codeleted	76	24	
Not codeleted	52	8	

*IDH*			0.082
Mutant	87	24	
Wildtype	41	8	

*Chemotherapy*			0.153
Yes	62	11	
No	66	21	

*Radiotherapy*			0.717
Yes	96	23	
No	32	9	

**Table 2:** Univariate and multivariate cox regression analyses of training groups.

Variables	Univariate analyses		Multivariate analyses	
	HR (95% CI)	*P* values	HR (95% CI)	*P* values
*Sex*		0.442	NA	
Male	Reference			
Female	1.284 (0.678–2.430)			

*Age (years)*		0.143	NA	
≥40	Reference			
<40	1.614 (0.851–3.062)			

*Preoperative KPS*		<0.001^*∗*^		<0.001^*∗*^
≥80	Reference		Reference	
<80	5.376 (2.831–10.206)		6.648 (2.184–20.231)	

*First presenting symptom*		<0.001^*∗*^		0.005^*∗*^
Seizures	Reference		Reference	
Others	7.069 (3.247–15.389)		3.711 (1.475–9.338)	

*Extent of resection*		<0.001^*∗*^		0.001^*∗*^
Gross total	Reference		Reference	
Nontotal resection or biopsy	8.504 (3.742–19.326)		5.521 (1.978–15.408)	

*Deep location*		0.023^*∗*^		0.217
Yes	Reference		Reference	
No	0.474 (0.249–0.904)		2.154 (0.637–7.288)	

*Gross tumor size*		0.003^*∗*^		0.009^*∗*^
≥6 cm	Reference		Reference	
<6 cm	0.381 (0.200–0.728)		0.391 (0.194–0.788)	

*Histology*		0.623	NA	
Astrocytoma	Reference			
Oligodendroglioma	0.852 (0.450–1.614)			

*1p19q*		0.623	NA	
Codeleted	Reference			
Not codeleted	0.852 (0.450–1.614)			

*IDH*		0.006^*∗*^		0.492
Mutant	Reference		Reference	
Wildtype	2.678 (1.322–5.425)		1.360 (0.565–3.273)	

*Chemotherapy*		0.609	NA	
Yes	Reference			
No	1.182 (0.623–2.241)			

*Radiotherapy*		<0.001^*∗*^		0.142
Yes	Reference		Reference	
No	3.567 (1.863–6.831)		0.366 (0.096–1.401)	

^*∗*^: *P* < 0.05; NA: not applicable.

**Table 3:** Detailed scores for all variables in nomograms.

Variables	Nomogram scores
*Preoperative KPS*	
≥80	0
<80	100

*First presenting symptom*	
Seizures	0
Others	68

*Extent of resection*	
Gross total	0
Nontotal resection or biopsy	91

*Gross tumor size*	
≥6 cm	58
<6 cm	0

*1p19q*	
Codeleted	0
Not codeleted	68

*IDH*	
Mutant	0
Wildtype	57

*Radiotherapy*	
Yes	0
No	67

*Chemotherapy*	
Yes	0
No	40

## Data Availability

The data used to support the findings of this study are available from the corresponding authors upon request.
